# Heat Extremes, Public Health Impacts, and Adaptation Policy in Germany

**DOI:** 10.3390/ijerph17217862

**Published:** 2020-10-27

**Authors:** Hans-Guido Mücke, Jutta Maria Litvinovitch

**Affiliations:** 1 Department of Environmental Hygiene, German Environment Agency (Umweltbundesamt/UBA), Corrensplatz 1, 14195 Berlin, Germany; 2 Unit ‘Health in Climate Change’, Federal Ministry for the Environment, Nature Conservation and Nuclear Safety (Bundesministerium für Umwelt, Naturschutz und nukleare Sicherheit/BMU), P.O. Box 120629, 53048 Bonn, Germany; jutta.litvinovitch@bmu.bund.de

**Keywords:** heat-related health impacts, mortality, morbidity, heat health warning system, heat health action plan, climate change, national adaptation strategy, Germany

## Abstract

Global warming with increasing weather extremes, like heat events, is enhancing impacts to public health. This essay focuses on unusual extreme summer heat extremes occurring in Germany at higher frequency, longer duration, and with new temperature records. Large areas of the country are affected, particularly urban settlements, where about 77% of the population lives, which are exposed to multiple inner-city threats, such as urban heat islands. Because harm to public health is directly released by high ambient air temperatures, local and national studies on heat-related morbidity and mortality indicate that vulnerable groups such as the elderly population are predominantly threatened with heat-related health problems. After the severe mortality impacts of the extreme summer heat 2003 in Europe, in 2008, Germany took up the National Adaptation Strategy on Climate Change to tackle and manage the impacts of weather extremes, for example to protect people’s health against heat. Public health systems and services need to be better prepared to improve resilience to the effects of extreme heat events, e.g., by implementing heat health action plans. Both climate protection as well as adaptation are necessary in order to be able to respond as adequate as possible to the challenges posed by climate change.

## 1. Introduction

Emerging literature suggests that global warming causes increasing weather extremes resulting in enhanced health risks for the current and future global population [1]. Health impacts of a changing climate are already present and are expected to be expanded and intensified in the future [1]. Climate change affects the environment and public health by direct impacts of weather extremes, such as heat, droughts, fires, and floods, as well as indirect effects like vector- and waterborne diseases even inducing changes in allergens, biodiversity, and ecosystems [2,3].

As recorded, particularly since the beginning of this millennium, global climate change with increasing ambient air temperature has led to more frequent, intense, and longer hot weather events in many regions throughout the world, including Germany [4,5,6,7]. Population health suffered already from increased morbidity and mortality during extreme heat periods, notably as recorded in summer 2003, which contributed Europe-wide to more than 70,000 premature deaths, about 7000 of which were in Germany [8,9,10]. Concerning heat exposure, Europe shows markedly higher vulnerability than Africa and Southeast Asia, very likely because of a higher elderly population living in urban agglomerations [11].

Concerns of increasing weather extremes induced by climate change are highlighted e.g., by the business-as-usual emission scenario, which projected for Germany an increase of the average temperature up to 1.3 °C by 2050 and 3.7 °C by 2100 compared to the period 1971–2000 [12]. Model simulations for Europe estimate that annual fatalities from extreme heat could rise from 2700 deaths/year today to about 30,000 to 50,000 by 2050 with the global warming of 1.5 °C and 2 °C, up to 90,000 Europeans with a warming of 3 °C, assuming present vulnerability to heat and without appropriate mitigation and adaptation measures [13]. Estimations of heat-related mortality in Europe have projected increasing effects considering the current climate model Representative Concentration Pathways (RCP 4.5 scenario “limiting greenhouse gas emissions” and RCP 8.5 “business as usual”) for this century [14]. Mitigation and adaptation responses to climate change lead to direct reductions in the burden of ill health, enhance community resilience, and address environmental and social inequalities [2,15]. 

This essay examines the trends of the increase of recent heat extremes in the 21st century and heat-related health impact studies in Germany. Further, it reports on the deployment to establish a policy frame on a national climate change adaptation strategy. The essay also provides discussion on the public health implications of intensifying heat events, on actions initiated by the government to support science investigations as well as administrative instruments, and on short- and long-term measures, such as heat health warning system and heat health action plan, needed to mitigate adverse heat health impacts and to improve public health protection.

## 2. Rising Course of Summer Heat Events—Going to Extremes

Ambient air temperature measurements were carried out by the German Meteorological Service (Deutscher Wetterdienst, DWD) since 1881. DWD continuously and extensively reports on weather and climate data [12]. Recent findings showed a long-term trend of rising temperature with more heat and less cold extremes [16]. The country-wide annual temperature mean value is 8.9 °C calculated for the international climatological reference period of 30 years from 1981 to 2010, with a linear increasing trend of 1.6 °C for the whole dataset (1881–2010). Global warming is distinctly being detected in Germany because nine out of the 10 warmest years occurred since the year 2000, reaching the maximum anomaly for the temperature mean value of 1.6 °C in 2018, as Table 1 shows (source: www.dwd.de/cdc).

Since the beginning of this century, unusual extreme summer heat events occurred in Germany more frequently, with new temperature records and of extraordinary long-lasting duration. Table 1 shows that 15 out of the last 20 summers (period from 2000 to 2019) the country-wide mean summer temperature calculated for the three months period June, July, and August exceeded the average value of 17.1 °C (reference period from 1981 to 2010), with a maximum anomaly of 2.6 °C in 2003. The 10 warmest summer periods appeared since 2002. Monthly peak anomalies have reached from 3.1 °C in August 2003 up to 4.0 °C in July 2006. In addition to that there is a significant increase of temperature records within last years. Six out of 10 records since 1881 had been measured since 2003 occurring both during short- or long-term heat extreme events (source: www.dwd.de/cdc), with a maximum of 42.6 °C in 2019.

Apart from increased mean and maximum temperature values the frequency and duration of extreme heat events have to be considered carefully for public health protection and prevention. Stable atmospheric high-pressure systems cause intensive summer heat extremes that periodically affect either the whole country or large areas in different regions, predominantly in topographically specific and densely populated areas like basins and valleys, such as the Rhine valley. As one example, the Rhine Main region is populated by around 5.7 million inhabitants. Part of this is the greater agglomeration area of Frankfurt/Main and surrounding cities (about 2.2 million inhabitants), which has been selected for an estimation to show a predominant urban “hot spot” case. 

The majority of DWD meteorological stations are remote. Only a limited number of monitoring sites are located in densely populated urban settings, which are most relevant for human exposure and heat-related health impact assessments. Currently, a general definition of lasting heat episodes, so-called heat waves, does not exist. To visualize the complexity of frequency, duration, and intensity of hot summers between 2001 and 2019, a minimum number of three consecutive days exceeding the 95th percentile (23.5 °C) of the annual temperature mean value (Tmean) has been calculated for the period 2001–2019 of the DWD measurement station Frankfurt/Main-Westend (Tmean 2001–2019: 11.7 °C; Tmean 1981–2010: 11.2 °C; Tmean 1961–1990: 10.6 °C; source: dwd.de). This site is characterized as suburban not far in distance from the city center.

**Figure 1:**
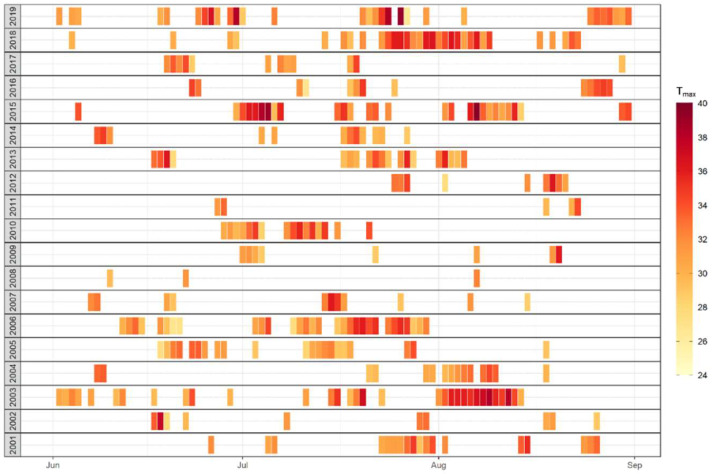
Daily air temperature maximum (Tmax in °C) on days during summer months exceeding the 95th percentile of the daily average air temperature calculated for the period 2001 to 2019 at the DWD station Frankfurt/Main-Westend.

Figure 1 depicts those extreme heat events for the summer months June, July and August between 2001 and 2019. Each bar is equal to one of these days; additionally, the color of each bar indicates the intensity of the maximum temperature (Tmax in °C). The figure shows that the bulk of heat events appeared in July, and the frequency of summer heat events may reach up to a maximum of five (2013) or six (2006) events. Partly, lasting periods are interrupted by short-term instabilities of the high-pressure system with such as a fresh air intrusion of one day only. Furthermore, the figure shows considerably long-lasting extreme heat events during summer 2003, 2006, and 2018, with the longest heat record of 18 consecutive days in July and August 2018. Heat events could be shorter, but stronger and hotter as experienced by each two single heat events of 2015 and 2019, with a temperature record at this DWD site of 40.2 °C on 25 July 2019.

## 3. Heat-Related Health Impacts—A New Emerging Issue for Public Health

Exposure to heat may directly cause serious human health problems with an increase in morbidity and mortality, e.g., by heat stress, heat exhaustion and heat stroke, heart attack and failure, and kidney disease due to dehydration [11,17]. Susceptible people most at risk, like elderly, outdoor workers, socially isolated population groups, and children, but also predisposed vulnerable groups with problems of the thermoregulatory system or cardiovascular or respiratory diseases [18]. During intense heat events population health suffers from increased morbidity and mortality, as recorded by the extreme heat period, so-called heat wave, of two weeks in August 2003 that induced exceptional effects on public health and to the health sector in mid and Western Europe and contributed Europe-wide to over 70,000 premature deaths, of which above 7000 took place in Germany [8,10]. The increase of atmospheric warming in Germany is estimated to face an average of approximately 8500 heat-related excess deaths per year by the end of this century [19].

Due to its demographic distribution, ageing population, high rates of urbanization and high prevalence of diabetes, cardiovascular and respiratory diseases, the population in Europe tend to be more and more at risk and highly vulnerable to heat exposure [3], features that are common for Germany too [20]. Heat vulnerability in Europe seems to be higher than in Africa and Southeast Asia, most probably because a large share of older population (42% older than 65 years) living in conurbation [11]. Furthermore, the vulnerability to people’s health depends on personal characteristics and constitution, individual behavior, societal and socioeconomic status, access to public health care and services, and levels of exposure. For example, people living in populated urban areas are primarily at higher health risks due to multiple exposure effects. Since 2015, more than half of the global population lives in urban agglomerations. Germany has a history of sustained urban population growth, reaching 77% in 2017 [21]. Due to various daily business activities such as transport, production, construction, and tourism, people living in urban settlements are exposed to multiple environmental threats from air, noise, water, and soil pollution; chemicals; and artificial light. In addition to that, during sunny hot summer days, buildings, streets, and surfaces absorb the energy of solar radiation and store thermal energy; this is the reason why the inner-city temperature does not cool down effectively during nighttime in comparison to suburban or rural areas. Known as urban heat island effect, it can reach to a temperature difference up to 10 °C [22,23,24]. The burden of persistently overheated inner cities stresses the thermoregulatory system of the human body for the whole day and shortens recovery at night [25,26]. 

As described before, in Germany, the frequency, intensity, and duration of extreme heat events increased in recent years considerably. Several public health studies have been investigated since the extreme summer heat event of 2003 showing evident impacts on heat related morbidity and mortality. The results of a few selected studies follow to give a brief overview, which is not intended to be complete, on respective investigations that have been conducted in Germany.

The adverse effects of high air temperature and heat events on human health had been studied at the national scale. A review indicated that extended periods between 2001 and 2010 of unusual heat stress induced significant increases in mortality on ischemic heart diseases, which is generally stronger for females than for males, and for chronic ischemic diseases than for myocardial infarctions [27]. Another part of the study analyzed simulations of several regional climate models forced by the moderate climate change scenario A1B. Three model time periods of 30 years were evaluated, representing present climate (1971–2000), near future climate (2021–2050), and remote future climate (2069–2098) [28]. Based on these model calculations, future heat events are projected to occur more often, be longer lasting, and be more dangerous. By the end of this century, the number of heat events is projected to double or triple compared to present climate, the average duration of heat episodes will increase by 30%, and the average number of hot days is expected to increase by 40 days per year on average, meaning that nearly every second summer day would lead to a substantial rise of the future heat-related health burden, particularly on the cardiovascular system [27,28]. 

A retrospective analysis on the mortality rates of ischemic heart diseases and chronic lower respiratory diseases, like asthma, bronchitis, and chronic lower respiratory diseases (CLRD) at the regional level in Germany for the period 2001–2015 has been conducted in combination with meteorological observations. Findings indicate that mortality and morbidity due to CLRD have a lower number of cases but show an increasing trend during hot periods. CLRD mortality increases by 19.9%, doubled as ischemic heart diseases mortality, which highlights the concerns of CLRD patients during hot days and extreme heat [29]. The results can help to take precautionary measures for respiratory patients, which are already available for cardiac patients in Germany. 

A study on total mortality estimated heat-related deaths between 2001 and 2015 in Germany on unusual expansion of environmental exposure situations due to heat extreme events country-wide, causing acute health impacts. The comparison of different indicators for heat exposure showed that the weekly mean temperature was most useful to explain the course of the weekly mortality during the summer with some variations between age groups and regions. The age groups (75–84) and (>85) were most affected. The maximum number of heat-related deaths was 7600 in 2003, followed by 6200 in 2006 and 6100 in 2015 [30]. The methods used in this investigation are foreseen for future national reporting services operated by the Robert Koch Institute (RKI).

On a local scale, the public health city authority of Frankfurt am Main conducted a study on severe heat-related morbidity as real-time surveillance via rescue service operations. In addition to mortality data, hospital admission, emergency calls, and department visit data of various heat-related diagnosis were used for the summer months (June, July, and August) from 2014 to 2018. Three heat events, defined of at least five consecutive days with a maximum temperature above 32 °C (perceived temperature, used for heat-related health alerts, so-called heat health warnings of the German Meteorological Service), occurred in 2015, 2016, and 2018, with a record duration of 18 consecutive days. The number of hospital admissions increased in July 2015 by 22%, respectively admissions of heat symptom diagnosis, such as heat exhausts, syncope or exsiccosis above 100%. The assessment of this noticeable long-lasting 2018 summer heat showed that an increasing duration of this extreme heat period triggered rising emergency rescue service operations due to total heat associated morbidity remained high with increased cases of exsiccosis and unclear fever [31,32]. 

Beside the increase of heat-related morbidity and mortality, heat stress conditions cause negative effects on the capability, effectivity and productivity of labor, as reported by the official German hospital statistics between 2007 and 2017 (http://de.statista.com). A number of peak cases (*n* = 2282) of heat strokes and exsiccosis occurred at work had been recorded for summer 2015. As reported by facts and figures of health insurances labor losses measured by “missing labor days” during summer months showed maximum numbers of about 42,500 in 2010 and 52,000 in 2015; generally, men are affected by two-thirds of all cases (www.gbe-bund.de). Such findings occurred in and are predominantly confirmed for other European countries [33].

## 4. From Science to Policy Achievements on Heat-Related Health Adaptation 

### 4.1. History of the General Policy Frame

First reports of the Intergovernmental Panel on Climate Change (IPCC) dating back to 2001 emphasized that adaptation to climate change is an important element when coping with global warming. However, this topic hardly received any attention because most of the actors involved concentrated on climate protection policies and measures both internationally and nationally. By now, it is mainly accepted that both climate protection and adaptation are necessary in order to be able to respond as adequately as possible to the challenges posed by climate change.

In 2005, the federal German policy took hold of the issue of adaptation to climate change for the first time to the Government’s Climate Protection programme, later to the Climate Action Programmes 2020 and 2030, as well as to the National Strategy for Sustainable Development based on the UN Sustainable Development Goals (SDGs) to reach the “Agenda 2030” aims [34]. Triggered by the exceptional heat event of summer 2003 in Europe the German Environment Agency (Umweltbundesamt, UBA) established the Competence Centre for Climate Change Impacts and Adaptation (KomPass) in 2006 to manage the implementation of actions to come. As the main input of their efforts, in December 2008, the German government adopted the National Adaptation Strategy on Climate Change (DAS) as a framework for policy decisions and action into force, containing 15 different fields of action, human health being the key issue [35]. The aim of DAS is to develop and initiate appropriate adaptation measures and instruments able to be applied for the prevention of climate change impacts to the society, environment, economy, and human health. In depth, it was recognized that a warming atmosphere induced increasing distinct weather extreme events, including heat extremes, in Germany since the beginning of this century with assigned health impacts to the population. In 2011, a first Adaptation Action Plan (DAS APA I, 2011–2015) had been implemented to turn DAS into action, including schemes and options for practical implementation, such as financial support, research fields, and public private partnerships [36]. The government supported adaptation concepts and community level projects between 2011 and 2017 with €9 million. A small proportion of only 10% was dedicated to the public health sector. 

Regarding the DAS field “human health,” due to the federal administrative system, a challenge was to initiate, coordinate, and manage regional public health adaptation actions in cooperation with the 16 German federal states (Bundesländer) considering their responsibilities, structures, and competence. Since 2008, internationally, Germany took a leading role to strengthen climate change-related health issues, which became one of the main pillars of the Fifth European Ministerial Conference on Environment and Health of the World Health Organization (WHO), taken place 2010 in Parma/Italy. The regional framework and commitment to act were appreciated by ministers of the WHO European Region. 

In 2012, and as part of APA I, an intersectoral Working Group on Public Health Adaptation to Climate Change had been set on behalf the Federal German Ministries of Environment and Health to establish a network for exchanging information, experiences and serving as advisors to the governmental process. National and state experts from science institutions and authorities from environment and public health sectors had been invited as board members to review research studies, assessments and pre-regulations for this new public health challenges and to give independent advice and guidance for policy decisions at the national level. In 2013, on behalf of this board, UBA and the Robert Koch Institute (RKI) jointly elaborated a guideline on climate change and health for politicians, stakeholders, and decision makers specifying adaptation concepts for prevention on communicable and non-communicable diseases, research, education, and risk communication, including the demand to develop a heat health action plan [37]. Building on this guideline, in 2013 a country-wide survey had been conducted to investigate status and deployment of adaptation tasks to climate-change-associated health risks. The study collected and analyzed data of about 330 actions at various scales. Selected best practice examples of potential health-related prevention and adaptation measures concerned UV radiation exposure, the spread of new invasive biota species, such as *Ambrosia artemisiifolia*, but emphasized distinctly that heat stress had been reported with greatest concern [38]. 

In response to DAS APA I [36], a cross-sectoral and consistent vulnerability assessment for Germany has been performed from 2011 to 2015, which served as a basis for the DAS progress report and the process to further develop a sustainable adaptation policy. The vulnerability assessment study on climate change was an interdisciplinary scientific task, which required the cooperation of different disciplines and bodies as well as the integration of regional and action field-specific expertise. Besides, the issue of heat extremes had been identified as cross-sectorial, relevant to other DAS action fields, such as agriculture, water, energy, transport, infrastructures, industries, tourism, and human health. It has been concluded that the impact of heat has high significance on human health at present, for which accelerated adaptation activities have to be initiated promptly [39].

### 4.2. First Adaptation Instruments and Measures

#### 4.2.1. Heat Health Warning Systems

Heat health warning systems (HHWS) are the main response to extreme heat events worldwide, alerting the general public and decision makers of dangerous heat–health situations. They consist of weather forecasts, methods to assess the weather–health relationship, a system of graded alerts and the communication of such alerts. Currently, 16 HHWS are in operation Europe-wide, most of them established after the 2003 extreme summer heat [33]. 

A European analysis of number and duration of heat waves (defined criteria: 98th percentile and a temperature threshold of 28 °C lasting of at least three days; dwd.de/rcc-cm) since 1950 shows a clear increasing trend. Specifically, within the last 70 years, nine out of 14 extreme heat waves with the longest duration (from seven up to 28 days) occurred since the beginning of this century [40].

The HHWS in Germany has been developed by the German Meteorological Service (Deutscher Wetterdienst, DWD) established shortly after the summer heat in 2003. Methodologically, the DWD HHWS is based on a perceived temperature model, calculates the heat load for regions and counties countrywide. Between April and October, short-term heat health warnings of the two stages “strong” and “extreme” and pre-heat information are issued when high levels of heat load are forecasted for at least two consecutive days. After receiving heat warnings public (health) authorities, clinics, and care homes then actively introduce and communicate respective adaptation measures via specific communication channels according to different levels of heat health warning. In 2017, the DWD HHWS had been supplemented by recommendations for vulnerable groups like the elderly and people living in densely populated areas [40]. 

Usually, due to regional climate zones in Germany, the lowest burden of heat stress appears in the northern part (Hamburg), the highest in the south-west (Freiburg), as shown explicitly for the heat extreme of 2006 and 2015, while in 2018 a maximum number of heat warnings were published for North and East Germany (Hamburg and Berlin). At the national level the total number of heat warnings ranged from 6418 in 2006, 6128 in 2015, to 5678 in 2018. The warnings show a countrywide maximum during the second half of July, as well as annual and regional variabilities. However, 15 years of DWD HHWS operation is not long enough to identify a trend of heat health warnings in Germany [40]. 

#### 4.2.2. Heat Health Action Plans

European countries, for example France [41], the Netherlands [42], and the United Kingdom [43], have successfully developed and established national heat health action plans (HHAP) shortly after the summer of 2003. Some of them are updated annually, and partly managed by the HHAP expert guidance document, which was published by the WHO Regional Office for Europe in 2008 [44], to shelter people against the impacts of extreme heat events. The main aim of a HHAP is to provide a harmonized common approach to prevent and reduce heat-related morbidity and mortality by issuing heat health warning alerts by a HHWS, to encourage planning in all relevant sectors, to integrate health into all policies and to raise awareness among the public, health, and social care workforce, as well as to mobilize resources for managing the effects of heat. In the context of the extreme summer heat of 2010 in Eastern Europe, the Federal Ministry for the Environment (BMU) conducted an international conference co-organized by the WHO, UBA, and DWD to review latest insight and lessons learned on climate change, extreme weather effects, and public health in Europe [45].

In 2016, as a core issue of the second Adaptation Action Plan (DAS APA II, 2016–2020) [46], the intersectoral Working Group on Public Health Adaptation to Climate Change took responsibility to develop governmental recommendations for the elaboration and implementation of a HHAP to protect human health in Germany, using the WHO HHAP guidance 2008 as masterminded blueprint. Public health prevention requires a portfolio of actions at different levels and time scales, which reaches from health system preparedness coordinated with meteorological early warning systems (HHWS) to timely public and medical advice and long-term improvements to housing and urban planning. Provisions should be integrated in a HHAP, including a communication plan, measures for the reduction of exposure, care for vulnerable population groups, and monitoring. Experiences from disaster risks scenarios of emergency and rescue services should be followed when planning for and responding to heat extremes. They should use and apply existing systems by a long-term approach and evaluation mechanisms. 

The recommendations for the development of a HHAP in Germany had been adopted and published by the government in 2017 [47]. They serve as a masterplan for the country to be applied at the levels of responsibility at federal states, counties, and municipalities. It is a consolidated approach for drawing up and establishing coordinated and applicable HHAP tailored to each region. Implementation will largely be carried out at the individual state or local level. Since its launch the HHAP recommendations were introduced and promoted countrywide to regional and local environment and public health bodies on several occasions. In 2019, a study has been carried out on German parliament databases (of the German Bundestag and the 16 federal states) that identified (i) the 2017 HHAP recommendations are well known at all state parliaments to date, but (ii) public health prevention and protection during heat periods are not a priority topic of parliamentary debates so far [48]. Since HHAP have not been systematically implemented at state or local levels in Germany, a number of sound examples of heat-related health prevention measures exist and had been published, such as an information flyer on individual behavior. However, it remains unclear whether these actions meet the criteria for effective medium and short-term protection [49]. 

After having experienced recent summer heat extremes of 2018 and 2019, there is a strong need to comprehensively implement HHAP at state and local levels, specifically targeting vulnerable population groups, and to evaluate and improve the effectiveness of existing tentative measures.

As the HHAP, for example, of France and Italy show, reporting and registering of heat-related excess mortality cases are carried out by computerized systems in cities and national statistic services, delayed by one to two days after a heat extreme had started. Such systems are extremely helpful and necessary for contemporary public health reactions, monitoring and evaluation. Because Germany has not developed such system yet, it is explicitly recommended in the DAS APA II monitoring report on adaptation to climate change [50] and foreseen to be turned into action in DAS APA III (2021–2025). A HHAP can be seen to strengthen climate change adaptation measures in other sectors as well, such as reduction of sealing soil surfaces, increasing green and blue spaces and inner-city ventilation channels through urban planning, which can contribute to abate urban heat island effects and thus to make cities and their population more resilient to the health impacts of heat in the future [47].

#### 4.2.3. The Local Project “HHAP for Elderly People of the City of Cologne”

Accordingly, based on the 2017 HHAP recommendations in 2019 in line with DAS APA II (2016–2020), a first city pilot project has been started to study the burden of heat-related health on elderly people living in different boroughs of Cologne, distinguished by demography, environment, culture and socio-economic structure, social networks, housing conditions, and exposure to urban heat islands. A personal interview survey had been performed at 258 households of elderly people (aged above 65 years) independently living alone. First results will be published soon. Further, in 2020, the study will investigate the heat-related health perception of people living in care homes. 

While the study results will be part of a HHAP primarily focused on the elderly population, the project aims to test its best measures applicable for the whole metropolitan community, and to derive appropriate adaptation and intervention options. The project is led by the city environment administration in cooperation, assistance, and support of other city administrative partners, e.g., from public health, planning, and transport and relevant stakeholders (https://www.stadt-koeln.de/artikel/67953/index.html). It is also part of a first national study that performs the efficiency of HHAP in Germany between 2019 and 2022.

There is high expectation of the National Adaptation Strategy on Climate Change (DAS), the Association of German Cities, as well as of respective town and community stakeholder organizations that the Cologne pilot project could be able to initiate a domino effect to other cities in the densely populated Rhine Ruhr Region of North Rhine Westphalia, as well as country-wide. 

## 5. Discussion

It has been clearly shown, as described above, that a global warming atmosphere triggers a rising trend of air temperature and unusual extreme summer heat events that directly harm human health in Germany, as recorded since the beginning of this century. Further, it is expected that the increase of temperature and the heat-related health burden will be a considerable challenge for next decades. Between 2000 and 2015, roughly 39,000 heat-related excess death cases had been estimated for Germany [30]. Heat-induced morbidity cases might even be higher, as a few local studies have indicated [31,32]. Due to evident heat-related health impacts and a few more effects of other expanding weather extremes, like droughts, heavy storms, and rain, climate-change-related health issues became more and more prominent in the society. Now, health professionals and the public health sector in Germany have identified this as an emerging issue for the future. To date, the exchange and inter-connections between health, environment, economy, energy, and climate sectors are not yet sufficiently considered, developed, or implemented in climate protection and adaptation policies.

It is important to know that the healthcare sector was responsible for approximately 2250 million tons of CO_2_ equivalent emissions in 2016, with a total of 4.6% of carbon emissions globally. In the same year, the well-established and accessible German healthcare sector accounted for about 70 million tons of CO_2_ equivalent emissions, corresponding to 5.2% of overall national emissions [3]. Healthcare services tend to be one main source of high emissions—for example, hospitals have great potential to reduce their energy consumption. Thus, this sector has considerable capabilities and is responsible to contribute to climate change mitigation, in line with the ambitions of the 2015 Paris Agreement to achieve carbon neutrality by 2050, responding to its mission to protect and promote human health [14]. Accordingly, in the currently ongoing project “Klik green,” about 250 hospitals participate in implementing low-investment energy saving measures to optimize logistics and food supply and to qualify climate clinic assists who develop mitigation goals and implement them [51].

Efforts to reduce greenhouse gas emissions as well as other environmental impacts of the healthcare sector are gaining momentum. For that reason, recently, various stakeholders and private and non-governmental organizations, expert associations, and climate activists brought up this issue on the agenda of environmentalists, medical doctors, and healthcare services in Germany to introduce new concepts and initiatives for climate protection and adaptation policies. With substantial governmental financial support abatement strategies offer substantial benefits for health at national and international scale, like less carbon fossil fuel combustion in the energy and transportation sectors with a positive side-effect of decreasing exposure to air pollution. Behavior and individual lifestyle decisions affect resource and energy demand and thus carbon emissions [52]. In the short term, influencing these decisions is an important cost-effective mitigation strategy often associated with positive health benefits. For example, as experiences from media campaigns (e.g., video spots at bus and train stops, establishing of new extra cycling lanes on busy streets) have shown the promotion of cycling and walking and the use of public transportation contributes to increase physical activity and to reduce air pollution. Both can substantially reduce the burden of noncommunicable diseases such as cardiovascular disease, cancer, diabetes, and chronic respiratory conditions, with considerable potential cost savings [53].

Heat events have been identified as one of the deadliest types of extreme weather events in Europe, causing tens of thousands of premature deaths [54]. It is anticipated that the situation will worsen with climate change, as global warming is expected to increase continuously the frequency, intensity and duration of heat waves significantly. Nevertheless, the adverse health impacts of hot weather and extreme heat events are largely preventable. From the climate change adaptation point of view Heat Health Warning Systems (HHWS) have been established internationally closely after the heat extremes of summer 2003, in Germany since 2005. HHWS are partly linked up. Germany demanded a communication network on heat health warnings in the WHO European Region. Preventing the consequences of heat extremes requires an array of actions at different levels, e.g., regarding the implementation of a Heat Health Action Plan (HHAP). Until today, more than 20 WHO European Member States subsequently developed national or subnational HHAP based on the WHO HHAP guidance [44] to avoid or reduce exposure, to communicate risks effectively, to take particular care of vulnerable population groups, and to manage mild and severe heat illness [54]. 

In Germany, governmental recommendations for HHAP had been developed to serve state and local authorities responsible for the implementation [47]. Since then, the HHAP document introduced, promoted, and discussed countrywide to environment and public health bodies on several occasions. Responses reveal that relevant stakeholders must be involved, near real-time surveillance and health data should be available, monitored and assessed, and human capacities at public health services have to be extended. In 2011, the WHO Regional Office for Europe published annexed information sheets to the 2008 WHO HHAP document for an appropriate HHAP implementation advising the general public, medical professionals and health services with guidance of best practice [54]. On request of the federal states, recently, this document has been translated into German for use by public health workforces at local level [55].

Although the 2017 HHAP recommendations and respective adaptation measures to prevent people from extreme heat events are well known and have been discussed at German state parliaments, they do not have priority yet. In spite of a significant number of reports and hearings on the heat-related health impacts of the extreme summer heat events particularly of 2003, 2006, 2010, 2015, 2018, and 2019, this is not considered as a priority issue of politician debates nor for concerted actions of public health bodies [48]. 

Aside from an anticipated implementation of HHAP in the near future, since summer 2003, a substantial number of single low-budget heat-related health preventive activities and measures had been established at vulnerable municipalities in Germany, such as public information material on the promotion of individual heat-related health prevention strategies to cope with hot weather and heat events [56]. Vulnerable groups, for example elderly people have a current proportion of 22% (2019) in the society. They are recognized to be at the highest heat-related health risk, especially those who are chronically ill, living isolated or confined to home. Care homes and institutions for the elderly and disabled are required to implement the 2017 HHAP recommendations, which include provisions for staff training, and in the medium term, building standards and the promotion of cooling centers. However, most of them who require nursing care receive it on a private visit at home from family members or outpatient home carers [20]. Therefore, the majority of such people at risk are not directly covered by HHAP recommendations, but advice for self-arranging a neighborhood “buddy system” is explicitly given. Concerning this, it will be interesting to see the results from the currently running pilot city HHAP project of Cologne and to follow which adaptation measures will be appropriate and proposed for sustainable implementation in the boroughs [57].

## 6. Conclusions

To conclude, it will be important to follow and learn from the first national study on the effectiveness and efficiency of the 2017 HHAP recommendations, which is going to be conducted between 2019 and 2022. Regional and local agencies must have given authority to evaluate the applicability and efficacy of HHAP operation and proposed intervention measures. In addition, they must be allowed to develop, implement, and evaluate pilot strategies and measures that can fit better for their local communities, and the measures that are found be successful can be integrated in the national HHAP strategy. Public health systems and services need to be better prepared to improve resilience to the effects of extreme heat events.

Within the governmental policy process of the 2008 National Adaptation Strategy on Climate Change (DAS), recent arrangements have been started to prepare the upcoming Adaptation Action Plan III (APA III, 2021–2025). As part of it, the intersectoral Working Group on Public Health Adaptation to Climate Change will continuously network, contact and exchange with partners at Federal State governments and local administrations to gain regular information about the progress and implementation of HHAP in general and about enduring heat-related health adaptation measures specifically. APA III recognizes the need to further investigate effective climate change adaptation policies and to improve the understanding of potential health co-benefits of mitigation measures, which is strongly requested by the 2019 Policy Brief for Germany of the Lancet Countdown on Health and Climate Change [58]. The scientific and public health community should strengthen communication on current and potential future climate change-related weather extremes, such as heat-related health impacts to raise awareness for building up resilience in the societies, as well as to motivate and inspire people to engage adaptive and preventive measures.

## Figures and Tables

**Table 1:** Ranking of the 10 warmest years in Germany since 1881 (temperature in °C).

Rank	Mean Temp. ^1^	Year	Mean s. Temp. ^2^	Year	Max. Temp. ^3^	Year
1.	10.5	2018	19.7	2003	42.6	2019
2.	10.3	2019	19.3	2018	40.3	2015
3.	10.3	2014	19.2	2019	40.2	2003
4.	9.9	2015	18.4	2015	40.2	1983
5.	9.9	2000	18.1	2006	39.6	1994
6.	9.9	2007	18.0	2002	39.6	1952
7.	9.7	1994	17.9	2017	39.0	2003
8.	9.6	2011	17.8	2016	38.9	1921
9.	9.6	2017	17.8	2010	38.8	2010
10.	9.6	2002	17.7	2013	38.6	2006

^1^ Mean temperature (1981–2010: 8.9 °C); ^2^ Mean summer temperature (June, July and August 1981–2010: 17.1 °C); ^3^ Maximum temperature (1881–2019).
